# When Machine Perfusion Falls Short: Identifying Risks of Liver Graft Failure After Normothermic Machine Perfusion

**DOI:** 10.1111/aor.70195

**Published:** 2026-07-18

**Authors:** Lauren E. Matevish, Abdullah K. Malik, Milana Harris, Ahmad Anouti, Taylor M. Coe, Simon Moosburner, Nathanael Raschzok, Arjmand Mufti, Parsia A. Vagefi, Madhukar S. Patel

**Affiliations:** ^1^ Department of Surgery, Division of Surgical Transplantation University of Texas Southwestern Medical Center Dallas Texas USA; ^2^ Department of HPB and Transplant Surgery, Freeman Hospital Newcastle Upon Tyne Hospitals NHS Foundation Trust Newcastle upon Tyne UK; ^3^ Newcastle Fibrosis Research Group Newcastle University Newcastle upon Tyne UK; ^4^ Department of Pediatrics University of Texas Southwestern Medical Center Dallas Texas USA; ^5^ Department of Surgery, Campus Charité Mitte Campus Virchow Klinikum, Charité, Universitätsmedizin Berlin, Corporate Member of Freie Universität Berlin and Humboldt‐Universität Zu Berlin Berlin Germany; ^6^ Biomedical Innovation Academy, BIH Charité Clinician Scientist Program, Berlin Institute of Health at Charité, Universitätsmedizin Berlin Berlin Germany; ^7^ Department of Internal Medicine, Division of Digestive and Liver Diseases University of Texas Southwestern Medical Center Dallas Texas USA

**Keywords:** graft failure, liver transplantation, normothermic machine perfusion, recipient selection, surgical complexity, transplant outcomes

## Abstract

**Background:**

Machine perfusion technology has redefined donor and recipient criteria for liver transplantation. Despite increasing adoption, data regarding graft and recipient factors associated with unsuccessful normothermic machine perfusion (NMP) outcomes remain limited.

**Methods:**

The Organ Procurement and Transplantation Network database was used to retrospectively identify adult patients undergoing deceased donor liver transplantation with NMP preservation between January 10, 2021, and December 31, 2024. Populations were stratified by 1‐year graft status, with donor and recipient factors compared. Logistic regression identified factors independently associated with NMP graft failure, and center analyses assessed program‐level impacts.

**Results:**

Among 4928 NMP cases, 398 (8.1%) experienced 1‐year graft failure. They were more frequently admitted to the intensive care unit (ICU) at transplant (21.1% vs. 10.6%, *p* < 0.001) and had more complex abdominal histories, including higher rates of TIPS(17.0% vs. 12.0%), prior abdominal surgery (64.1% vs. 54.7%), and prior liver transplant (10.3% vs. 3.4%) (all *p* < 0.01). Regression analysis identified increased odds of graft failure with MELD score 35 (aOR 2.10 [1.21–3.63]), low recipient functional status (aOR 2.05 [1.31–3.21]), and prior abdominal surgery (aOR 1.33 [1.01–1.76]). Alcohol‐associated liver disease (aOR 0.36 [0.23–0.56]) was the only factor conferring a protective effect. Centers with higher‐than‐expected standardized NMP graft failure rates were higher‐volume programs with low‐risk case mixes; no centers had persistently high standardized failure rates throughout the study period.

**Conclusion:**

Recipient factors, particularly those conferring surgical complexity, are more significantly associated with NMP graft failure than donor factors. Despite increased adoption of perfusion technologies to mitigate donor and operative risk, transplant provider clinical judgment remains critical for optimizing recipient‐donor matching and outcomes.

## Graphical abstract

Analysis of 4928 normothermic machine perfusion liver transplants reveals that recipient factors, particularly MELD ≥ 35, surgical complexity, ICU status, and functional impairment are stronger predictors of graft failure than donor characteristics, emphasizing the continued importance of clinical judgment in patient selection.

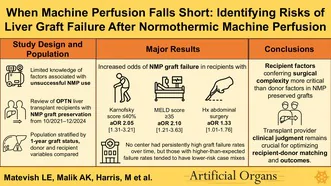

**Abbreviations**
ALD: alcohol‐associated liver disease
BMI: body mass index
CIT: cold ischemia time
DBD: donation after brain death
DCD: donation after cardiac death
HTN: hypertension
ICU: intensive care unit
IVDU: intravenous drug use
LT: liver transplant
MASLD: metabolic dysfunction‐associated steatotic liver disease
MELD: Model for End Stage Liver Disease
NMP: normothermic machine perfusion
PNF: primary nonfunction
SCS: static cold storage
SMR: standardized mortality rate
TIPS: transjugular intrahepatic portosystemic shunt

## 1 Introduction

Liver transplantation (LT) is the only available treatment for patients with end‐stage liver disease, which accounts for over 50 000 deaths in the United States (U.S.) annually [1]. However, LT is critically limited by the availability of donor organs. Normothermic machine perfusion (NMP), a technology developed to improve organ preservation, reduce ischemic graft injury, and permit functional assessment prior to transplant, has helped increase organ utilization and graft availability for transplant [2]. Since its approval by the U.S. Food and Drug Administration in September 2021, NMP has disrupted the standard of care for liver preservation, further changing conventional organ acceptance criteria, particularly for donation after circulatory death (DCD) grafts [3].

Most of the published literature has focused on the numerous short‐term benefits of NMP, including lowering organ discard rates [4, 5, 6], prolonging the acceptable duration of preservation [6, 7], reducing the risk of ischemia reperfusion injury [5, 8], and decreasing rates of post‐perfusion syndrome [9, 10] and early allograft dysfunction [11]. While randomized studies demonstrate equivalent patient and graft outcomes compared to SCS [12, 13], these trials employed strict inclusion criteria selecting predominantly low‐risk organs, with limited representation of DCD and extended criteria donors that now constitute the primary indication for NMP in clinical practice [5, 6]. Several subsequent retrospective studies have noted improved short‐term graft survival of livers preserved with NMP compared to SCS [7, 11], especially in older livers [14] and those from DCD donors [11, 15]. Specifically, NMP use has led to lower reported rates of graft failure due to primary nonfunction (PNF) [4], ischemic cholangiopathy [4], and hepatic artery thrombosis [4]. Indeed, the greatest benefit from NMP appears to arise from its use for preservation of these marginal organs (DCD donors, older donors, etc.) [10, 16], as it additionally allows for organ viability assessment prior to transplantation [2, 17].

Despite these benefits and the rapid adoption of NMP at programs across the country, the limits of this technology and its best uses remain underexplored. Beyond the nonspecific category of marginal grafts, there are no standard criteria for when NMP should be used, which donors or recipients receive the most benefit, or when it may be futile. Due to the significant costs associated with NMP, especially when compared to static cold storage (SCS), understanding where it provides minimal subsidy to recipient outcomes can help providers make decisions regarding its utilization. We aimed to assess the rate of graft failure in the NMP cohort over the three‐year period since mainstream adoption and characterize recipient and donor factors associated with futile use of NMP, defined by 1‐year graft failure. Additionally, we assessed the contribution of center variation in NMP graft failure rates and changes in these rates over time, as well as recipient and donor characteristics at high fail rate centers.

## 2 Methods

### 2.1 Data Source and Population

The Organ Procurement and Transplantation Network (OPTN) Standard Transplant Analysis and Research (STAR) files were used to identify all patients undergoing deceased donor liver transplant between October 1, 2021 and December 31, 2024. Cases were included if the donor liver was preserved using NMP, as documented directly within the database. Those utilizing other types of machine perfusion, including hypothermic machine perfusion, were excluded. Pediatric recipients and recipients of split liver transplants were also excluded. Patients were then stratified into groups based on graft status at 1 year (i.e., graft failure/survival). Graft failure was defined as a non‐functioning graft at day 365 posttransplant, either due to graft failure and retransplantation or patient death. Recipient and donor factors were compared between groups. This study was reviewed by the institutional IRB and determined to be exempt.

Recipient variables included demographic and clinical information, laboratory Model for End Stage Liver Disease (MELD) score, primary liver diagnosis, diagnosis of hepatocellular carcinoma, time on the liver transplant waitlist, Karnofsky functional status at transplant, admission status prior to transplant (intensive care unit (ICU) admit, hospital admit, not hospitalized), prior abdominal surgery, prior liver transplant, existing transjugular intrahepatic portosystemic shunt (TIPS), portal venous thrombus, and multiorgan transplant. Deceased donor factors included demographics, cause of death, donor type, terminal AST, ALT, and total bilirubin, graft macrosteatosis percentage and liver biopsy fibrosis scoring (if biopsy performed), total preservation time (documented as cold ischemia time [CIT]), donor medical history of diabetes or hypertension, history of IV drug use, heavy alcohol use (i.e., ≥ 2 drinks/day), and any clinical infection prior to donation.

### 2.2 Statistical Analysis

For univariate analyses, a comparison of continuous variables was performed using the Mann–Whitney U test. The Pearson chi‐squared coefficient was applied to categorical and binary data elements. Median values with interquartile ranges [IQR] were reported for nonparametric continuous variables. Logistic regression modeling was performed to identify recipient and donor factors associated with risk of 1‐year graft failure in all NMP cases; separate models were then evaluated for donation after brain death (DBD) NMP and DCD NMP grafts. Variables were included if significant on univariate analyses (i.e., *p* < 0.05), or deemed to be clinically relevant, and included recipient age, recipient sex, laboratory MELD score (stratified as < 15, 15–24, 25–34, and ≥ 35), liver diagnosis, Karnofsky functional status at transplant, hospitalization status, portal venous thrombus, prior abdominal surgery, prior liver transplant, TIPS, donor age, donor body mass index (BMI), DCD donor, donor total bilirubin, preservation time, and donor history of hypertension and diabetes.

Graft failure rates at 30 days, 90 days, 1 year, and 2 years were compared between NMP and SCS‐preserved grafts, with causes of failure further characterized between groups. Kaplan–Meier curves were generated to compare graft survival between groups. Sub‐analyses within the NMP cohort examined graft survival stratified by donor type (DCD vs. DBD) and by recipient MELD category.

### 2.3 Center‐Level Graft Failure Analysis

Funnel plots assessed systematic heterogeneity across centers, plotting institutional standardized graft mortality rates (SMR) against the expected number of 1‐year NMP graft failure events; graphs were centered on 1, in which observed graft failure events equaled the expected number of events. Institutional graft failure rates were adjusted for case mix, including recipient age, recipient sex, laboratory MELD score, portal venous thrombus, prior liver transplant, TIPS, donor age, DCD donor, donor total bilirubin, and donor history of hypertension. Graphs for all NMP cases were plotted, and outliers were identified. To further address changes over time in graft failure rates which may arise due to increased NMP adoption and institutional experience, cases were stratified into three time periods of equal length: (A) January 1, 2021–December 31, 2022, (B) January 1, 2023–December 31, 2023, and (C) January 1, 2024–December 31, 2024. Outlier centers with higher‐than‐expected NMP graft failure rates over the study period were identified; recipient and donor characteristics at these centers were compared to those at all other centers.

All analyses were performed using Stata 18 (StataCorp, College Station, TX), and a *p*‐value of < 0.05 was considered statistically significant. This study was conducted and reported in accordance with the Strengthening the Reporting of Observational Studies in Epidemiology (STROBE) guidelines.

## 3 Results

Over the study period, 398 cases of 1‐year graft failure (8.1%, total *N* = 4928) after NMP preservation were identified; 8.1% of SCS cases during the same time had graft failure at 1 year (*N* = 2020/24787; *p* = 0.86). When stratified by donor type, 1‐year failure was 9.9% for DBD NMP transplants (*N* = 208/2107), and 6.7% for DCD NMP transplants (*N* = 190/2821; *p* < 0.001).

NMP recipients with graft failure were largely demographically similar to those surviving in terms of age, sex, ethnicity, and BMI (Table 1). Clinically, they more often had a primary diagnosis of metabolic dysfunction‐associated steatotic liver disease (MASLD; 24.1% vs. 20.3%) or malignancy (22.5% vs. 18.3%), while a lower proportion had alcohol‐associated liver disease (ALD) (19.1% vs. 34.7%) (*p* < 0.001). Laboratory MELD scores were slightly higher in the graft failure group (22 [16–32] vs. 21 [15, 16, 17, 18, 19, 20, 21, 22, 23, 24, 25, 26, 27], *p* < 0.001) as well. Failures were more commonly seen in recipients who were in the ICU (21.1% vs. 10.6%) or hospitalized (21.4% vs. 18.5%) at time of transplant (*p* < 0.001), and in those with lower Karnofsky functional scores (score 10%–40%: 44.7% vs. 30.0%; score 80%+: 14.9% vs. 22.0%; *p* < 0.001). Additionally, recipients with graft failure had more complex abdominal histories, including higher rates of TIPS (17.0% vs. 12.0%, *p* < 0.01), prior abdominal surgery (64.1% vs. 54.7%, *p* < 0.001), or prior liver transplant (10.3% vs. 3.4%, *p* < 0.001).

**TABLE 1:** Demographic and clinical characteristics of patients undergoing liver transplant with a graft preserved with normothermic machine perfusion, stratified by graft status at 1 year.

	Graft survival (*N* = 4530)	Graft failure (*N* = 398)	Total (*N* = 4928)	*p*
Recipient age (years)	58 [49–65]	60 [51–66]	58 [49–65]	0.13
Recipient sex (male)	63.2%	58.5%	62.8%	0.06
Recipient ethnicity				0.06
White	3073 (67.8%)	276 (69.3%)	3349 (68.0%)	
Black	220 (4.9%)	31 (7.8%)	251 (5.1%)	
Hispanic	895 (19.8%)	65 (16.3%)	960 (19.5%)	
Asian	169 (3.7%)	12 (3.0%)	181 (3.7%)	
Recipient BMI (kg/m^2^)	28.7 [25.0–32.9]	28.9 [24.8–33.4]	28.7 [24.9–32.9]	0.36
Recipient lab MELD	21 [15–27]	22 [16–32]	21 [15–28]	**< 0.001**
Primary diagnosis				**< 0.001**
MASLD	901 (20.3%)	91 (24.1%)	992 (20.6%)	
ALD	1543 (34.7%)	72 (19.1%)	1615 (33.5%)	
Viral	209 (4.7%)	16 (4.2%)	225 (4.7%)	
Malignancy	813 (18.3%)	85 (22.5%)	898 (18.6%)	
Cholestatic	310 (7.0%)	22 (5.8%)	332 (6.9%)	
Metabolic	75 (1.7%)	11 (2.9%)	86 (1.8%)	
Autoimmune	106 (2.4%)	13 (3.4%)	119 (2.5%)	
Unknown/cryptogenic	161 (3.6%)	13 (3.4%)	174 (3.6%)	
Other	327 (7.4%)	54 (14.3%)	381 (7.9%)	
HCC diagnosis	22.4%	23.4%	22.5%	0.66
Time on waitlist (days)	52.5 [12–212]	50 [12–221]	52 [12–213]	0.57
Karnofsky functional status				**< 0.001**
10%–40%	1336 (30.0%)	177 (44.7%)	1513 (31.2%)	
50%–70%	2133 (48.0%)	160 (40.4%)	2293 (47.3%)	
80%+	978 (22.0%)	59 (14.9%)	1037 (21.4%)	
Admission status at transplant				**< 0.001**
ICU	482 (10.6%)	84 (21.1%)	566 (11.5%)	
Hospitalized	839 (18.5%)	85 (21.4%)	924 (18.8%)	
Not hospitalized	3209 (70.8%)	229 (57.5%)	3438 (69.8%)	
Prior liver transplant	155 (3.4%)	41 (10.3%)	196 (4.0%)	**< 0.001**
Portal venous thrombus	689 (15.3%)	69 (17.4%)	758 (15.4%)	0.26
Prior abdominal surgery	2437 (54.7%)	252 (64.1%)	2689 (55.5%)	**< 0.001**
TIPS	521 (12.0%)	65 (17.0%)	586 (12.4%)	**< 0.01**
Multiorgan transplant	410 (9.1%)	51 (12.8%)	461 (9.4%)	**0.01**
Graft failure due to cholangiopathy	NA	18 (4.5%)	18 (0.4%)	NA
Graft failure due to PNF	NA	39 (9.8%)	39 (0.8%)	NA
Retransplanted	NA	104 (26.1%)	104 (2.4%)	NA
1‐year patient mortality	NA	294 (73.9%)	294 (6.0%)	NA

*Note:* The bold values indicate the statistical significance of *p* < 0.05.

Abbreviations: ALD, alcohol‐associated liver disease; BMI, body mass index; HCC, hepatocellular carcinoma; ICU, intensive care unit; MASLD, Metabolic dysfunction‐associated steatotic liver disease; MELD, Model for End Stage Liver Disease; PNF, primary nonfunction; TIPS, transjugular intrahepatic portosystemic shunt.

With regards to donors, the graft failure group had lower rates of DCD donors compared to the survival group (47.4% vs. 58.1%, *p* < 0.001) (Table 2). Failed grafts also came from donors with higher rates of hypertension (51.8% vs. 44.5%, *p* < 0.01) and diabetes (22.0% vs. 17.4%, *p* = 0.02). Overall preservation time was longer in the failure group (16.3 [11.9–20.3] hours vs. 15.1 [11.4–19.1], *p* < 0.001). No differences were seen between groups in donor age, sex, BMI, ethnicity, cause of death, terminal laboratory AST or ALT, or biopsy findings.

**TABLE 2:** Donor demographic and clinical characteristics of grafts preserved with normothermic machine perfusion, stratified by 1‐year graft status.

	Graft survival (*N* = 4530)	Graft failure (*N* = 398)	Total (*N* = 4928)	*p*
Donor age (years)	48 [35–59]	50 [36–589]	48 [35–59]	0.52
Donor sex (male)	60.9%	56.5%	60.6%	0.08
Donor BMI (kg/m^2^)	28.3 [24.2–33.4]	27.8 [24.6–33.1]	28.3 [24.2–33.3]	0.52
Donor ethnicity				0.39
White	3023 (66.7%)	253 (63.6%)	3276 (66.5%)	
Black	656 (14.5%)	59 (14.8%)	715 (14.5%)	
Hispanic	627 (13.8%)	64 (16.1%)	691 (14.0%)	
Asian	129 (2.8%)	14 (3.5%)	143 (2.9%)	
Cause of death				0.33
Anoxia	2313 (51.1%)	187 (47.0%)	2500 (50.7%)	
Stroke	1155 (25.5%)	120 (30.2%)	1275 (25.9%)	
Head trauma	886 (19.6%)	74 (18.6%)	960 (19.5%)	
DCD donor	2631 (58.1%)	190 (47.4%)	2821 (57.2%)	**< 0.001**
Terminal AST	46 [26–82]	42 [25–77]	45 [26–82]	0.28
Terminal ALT	37 [21–73]	35 [20–65]	37 [21–72]	0.37
Terminal total bilirubin	0.6 [0.4–0.9]	0.6 [0.4–0.9]	0.6 [0.4–0.9]	**0.02**
Preservation time^a^	15.1 [11.4–19.1]	16.3 [11.9–20.3]	15.2 [11.5–19.2]	**< 0.001**
Macro fat (%)	5.0 [0.0–10.0]	5.0 [0.0–10.0]	5.0 [0.0–10.0]	0.30
Liver biopsy fibrosis				0.98
None	1764 (73.5%)	164 (74.5%)	1928 (73.6%)	
Some portal	485 (20.2%)	41 (18.6%)	526 (20.1%)	
Most portal	118 (4.9%)	12 (5.5%)	130 (5.0%)	
Most with bridging	29 (1.2%)	3 (1.4%)	32 (1.2%)	
Incomplete cirrhosis	1 (0.0%)	0 (0.0%)	1 (0.0%)	
Donor diabetes	775 (17.4%)	87 (22.0%)	862 (17.8%)	**0.02**
Donor HTN	1980 (44.5%)	205 (51.8%)	2185 (45.1%)	**< 0.01**
Donor IVDU use	462 (10.5%)	34 (8.6%)	496 (10.3%)	0.25
Donor EtOH use	921 (20.3%)	73 (18.3%)	994 (20.2%)	0.34
Donor clinical infection	3517 (77.7%)	301 (75.6%)	3818 (77.6%)	0.33

*Note:* The bold values indicate the statistical significance of *p* < 0.05.

Abbreviations: BMI, body mass index; CIT, cold ischemia time; DCD, donation after circulatory death; EtOH, alcohol; HTN, hypertension; IVDU, intravenous drug use; NRP, normothermic regional perfusion.

^a^ Preservation time includes all time from the cold flushing of the donor organ until the graft is removed from the preservation method to be implanted into the recipient, including both SCS and machine perfusion time.

In recipients with a documented cause of graft failure in the year after NMP transplant, no significant differences were seen in etiology compared to SCS graft failures (Table 3). PNF (NMP 9.8% vs. SCS 8.6%, *p* = 0.45) was the primary recorded cause for graft failure in both groups; when contextualized within the entire study cohort, overall PNF rates remained comparable between groups at 0.8% in NMP and 0.7% in SCS recipients. Hepatic artery thrombosis was the second most common cause in both groups (NMP 7.5% vs. SCS 7.0%, *p* = 0.72), while diffuse cholangiopathy ranked third in NMP recipients (4.5% vs. SCS 4.6%, *p* = 0.94) compared to infection in SCS recipients (NMP 4.3% vs. SCS 6.1%, *p* = 0.15).

**TABLE 3:** Rates of graft failure between grafts preserved with static cold storage (SCS) versus normothermic machine perfusion (NMP), and in the NMP cohort, between donation after brain death (DBD) and donation after circulatory death (DCD) cohorts. Documented etiologies of 1‐year graft failure are further described between SCS and NMP cohorts.

	SCS *N* = 24 787 (83.4%)	NMP *N* = 4928 (16.6%)	Total *N* = 29 715	*p*
30‐day graft failure	702 (2.8%)	141 (2.9%)	843 (2.8%)	0.91
90‐day graft failure	1190 (4.8%)	231 (4.7%)	1421 (4.8%)	0.73
1‐year graft failure	2020 (8.1%)	398 (8.1%)	2418 (8.1%)	0.86
2‐year graft failure	1941 (11.0%)	204 (10.5%)	2145 (11.0%)	0.52

	NMP DBD *N* = 2107 (42.8%)	NMP DCD *N* = 2821 (57.2%)	Total *N* = 4928	*p*
30‐day graft failure	85 (4.0%)	56 (2.0%)	141 (2.9%)	**< 0.001**
90‐day graft failure	129 (6.1%)	102 (3.6%)	231 (4.7%)	**< 0.001**
1‐year graft failure	208 (9.9%)	190 (6.7%)	398 (8.1%)	**< 0.001**
2‐year graft failure	102 (11.5%)	102 (9.7%)	204 (10.5%)	0.22

	SCS *N* = 2020 (83.5%)	NMP *N* = 398 (16.5%)	Total *N* = 2418	*p*
Primary nonfunction	174 (8.6%)	39 (9.8%)	213 (8.8%)	0.45
Hepatic artery thrombosis	142 (7.0%)	30 (7.5%)	172 (7.1%)	0.72
Diffuse cholangiopathy	93 (4.6%)	18 (4.5%)	111 (4.6%)	0.94
Infection	124 (6.1%)	17 (4.3%)	141 (5.8%)	0.15
Chronic rejection	25 (1.2%)	5 (1.3%)	30 (1.2%)	0.98
Other vascular thrombosis	34 (1.7%)	7 (1.8%)	41 (1.7%)	0.92
Acute rejection	43 (2.1%)	7 (1.8%)	50 (2.1%)	0.64
Other	14 (0.7%)	6 (1.5%)	20 (0.8%)	0.10

*Note:* The bold values indicate the statistical significance of *p* < 0.05.

On regression analysis, a primary diagnosis of ALD was identified as a protective factor (aOR 0.36 [0.23–0.56]) (Table 4). Complex recipient factors, including low Karnofsky score (10%–40%, aOR 2.05 [1.31–3.21]), MELD score ≥ 35 (aOR 2.10 [1.21–3.63]), and prior abdominal surgery (aOR 1.33 [1.01–1.76]) were all associated with an increased risk of graft failure. Graft preservation time also had a significant aOR over 1. For DBD grafts specifically, pretransplant ICU admission (aOR 2.24 [1.12–4.47]) and TIPS (aOR 1.93 [1.15–3.21]) were also associated with graft failure. The only unique risk factors in the DCD population were a recipient diagnosis of metabolic disease (aOR 4.33 [1.17–15.96]) and a donor medical history of hypertension (aOR 1.80 [1.16–2.79]).

**TABLE 4:** Logistic regression of recipient and donor factors associated with risk of 1 year graft failure in all cases utilizing normothermic machine perfusion (NMP), in DBD cases using NMP, and in DCD cases using NMP.

	All NMP cohort		NMP DBD cohort		NMP DCD Cohort	
	Odds ratio [95% CI]	*p*	Odds ratio [95% CI]	*p*	Odds ratio [95% CI]	*p*
Recipient age (years)	1.00 [0.99–1.02]	0.70	1.01 [1.00–1.03]	0.14	0.99 [0.97–1.01]	0.19
Recipient sex (male)	0.82 [0.62–1.09]	0.18	0.76 [0.51–1.14]	0.19	0.90 [0.60–1.34]	0.59
Recipient lab MELD						
< 15	Reference					
15–24	1.04 [0.72–1.51]	0.84	0.92 [0.50–1.68]	0.78	1.11 [0.68–1.81]	0.67
25–34	1.19 [0.81–1.75]	0.37	1.25 [0.68–2.31]	0.48	1.11 [0.67–1.83]	0.69
> 34	2.10 [1.21–3.63]	**0.01**	2.05 [1.00–4.23]	0.05	2.93 [1.08–7.93]	0.04
Primary diagnosis						
MASLD	Reference					
ALD	0.36 [0.23–0.56]	**< 0.001**	0.31 [0.16–60]	**< 0.001**	0.39 [0.22–0.72]	**< 0.001**
Viral	0.92 [0.48–1.74]	0.79	1.12 [0.46–2.71]	0.80	0.71 [0.26–1.91]	0.49
Malignancy	1.40 [0.95–2.07]	0.09	1.69 [0.92–3.10]	0.09	1.24 [0.73–2.09]	0.43
Cholestatic	0.88 [0.49–1.59]	0.68	0.97 [0.41–2.31]	0.95	0.74 [0.32–1.72]	0.49
Metabolic	1.71 [0.67–4.37]	0.26	4.33 [1.17–15.96]	**0.03**	0.40 [0.05–3.08]	0.38
Autoimmune	1.15 [0.50–2.61]	0.75	0.56 [0.15–2.08]	0.38	1.54 [0.52–4.56]	0.43
Unknown/cryptogenic	0.85 [0.41–1.75]	0.66	0.64 [0.22–1.82]	0.40	0.97 [0.35–2.65]	0.95
Other	1.32 [0.78–2.21]	0.30	1.39 [0.70–2.75]	0.35	1.09 [0.44–2.68]	0.85
Karnofsky status						
80%–100%	Reference					
50%–70%	1.32 [0.91–1.91]	0.14	1.34 [0.73–2.45]	0.35	1.37 [0.85–2.20]	0.20
10%–40%	2.05 [1.31–3.21]	**< 0.001**	2.17 [1.08–4.35]	**0.03**	2.00 [1.09–3.69]	**0.03**
Admission status at transplant						
Not hospitalized	Reference					
Hospitalized	1.18 [0.77–1.81]	0.44	1.57 [0.83–2.94]	0.17	1.11 [0.60–2.05]	0.75
ICU	1.43 [0.84–2.45]	0.19	2.24 [1.12–4.47]	**0.02**	0.24 [0.05–1.02]	0.05
Portal venous thrombus	1.00 [0.70–1.41]	0.98	0.71 [0.41–1.24]	0.23	1.36 [0.86–2.16]	0.19
Prior abdominal surgery	1.33 [1.01–1.76]	**0.046**	1.67 [1.10–2.53]	**0.02**	1.07 [0.72–1.59]	0.75
TIPS	1.42 [0.99–2.04]	0.06	1.93 [1.15–3.21]	**0.01**	1.03 [0.59–1.78]	0.92
Prior transplant	1.33 [0.70–2.52]	0.39	0.98 [0.45–2.41]	0.95	2.96 [0.83–10.53]	0.09
Donor age	1.01 [0.99–1.012]	0.23	1.01 [1.00–1.03]	0.08	1.00 [0.98–1.01]	0.71
Donor BMI	1.00 [0.98–1.01]	0.63	0.99 [0.96–1.01]	0.29	1.00 [0.98–1.03]	0.82
DCD donor	0.81 [0.61–1.08]	0.16	NA		NA	
Terminal total bilirubin	1.15 [0.98–1.34]	0.09	1.17 [0.98–1.41]	0.09	1.20 [0.87–1.66]	0.27
Preservation time^a^	1.03 [1.01–1.05]	**< 0.01**	1.02 [0.98–1.05]	0.32	1.04 [1.01–1.08]	**0.01**
Donor diabetes	1.24 [0.89–1.72]	0.21	1.60 [0.98–2.59]	0.06	1.05 [0.65–1.69]	0.85
Donor HTN	1.31 [0.97–1.78]	0.08	0.92 [0.59–1.45]	0.73	1.80 [1.16–2.79]	**0.01**

*Note:* The bold values indicate the statistical significance of *p* < 0.05.

Abbreviations: ALD, alcohol‐associated liver disease; BMI, body mass index; DCD, donation after circulatory death; HTN, hypertension; ICU, intensive care unit; MASLD, Metabolic dysfunction‐associated steatotic liver disease; MELD, Model for End Stage Liver Disease; NRP, normothermic regional perfusion; TIPS, transjugular intrahepatic portosystemic shunt.

^a^ Preservation time includes all time from the cold flushing of the donor organ until the graft is removed from the preservation method to be implanted into the recipient, including both SCS and machine perfusion time.

Kaplan Meier graphs of overall graft survival demonstrated similar rates between NMP and SCS cohorts (Figure 1A); rates of graft failure were not significantly different between SCS and NMP cohorts at interval time points from 30 days to 2 years (Table 3). On analysis of the NMP survival curves, DCD NMP‐preserved grafts showed improved survival compared to DBD grafts, followed by convergence around 800 days (Figure 1B). Indeed, DCD NMP graft failure rates were significantly lower at 30 days (2.0% vs. 4.0%, *p* < 0.001), 90 days (3.6% vs. 6.1%, *p* < 0.001), and 1 year (6.7% vs. 9.9%, p < 0.001) compared to DBD NMP grafts, although failure rates at 2 years were similar (9.7% vs. 11.5%, *p* = 0.22) (Table 3). When stratified by MELD scores, NMP graft survival did not differ significantly between recipients with MELD scores < 15, 15–25, or 25–35 (Figure 1C; all *p* > 0.05). However, recipients with MELD ≥ 35 demonstrated significantly worse NMP graft survival compared to all lower strata (30 days: 6.4% vs. 2.6% vs. 2.5% vs. 2.5%; 90 days: 11.2% vs. 4.6% vs. 3.5% vs. 3.8%; 1 year: 15.1% vs. 7.7% vs. 6.8% vs. 7.4%; all *p* < 0.001).

**FIGURE 1:**
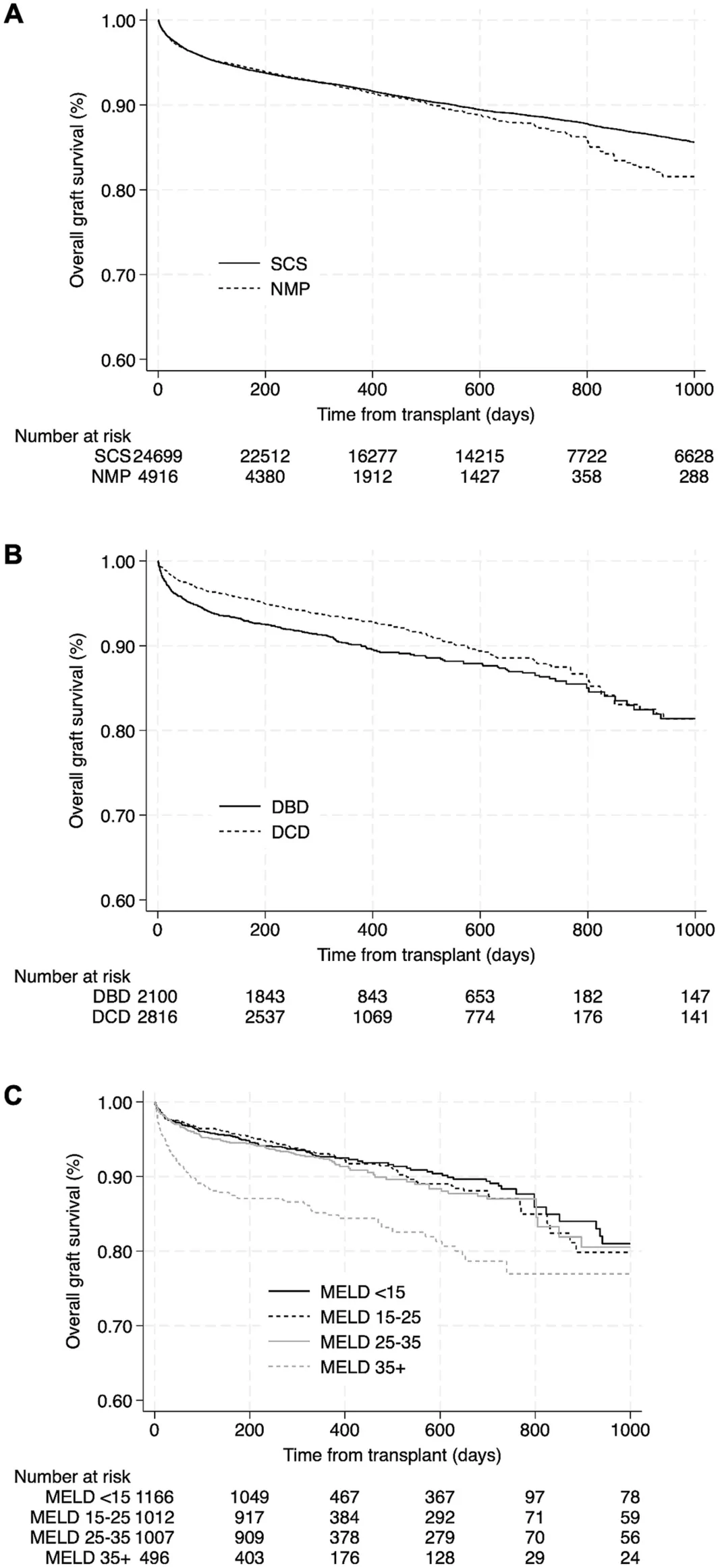
Kaplan–Meier curves of overall graft survival between recipients undergoing LT with (A) grafts preserved with SCS vs. NMP, (B) NMP‐preserved grafts from DBD vs. DCD donors, and (C) NMP‐preserved grafts at varying recipient MELD strata.

### 3.1 Center‐Level Analysis

Due to heterogeneous acceptance criteria, transplant practices, and adoption curves across institutions, we further examined NMP failure rates at the center‐level to explore individual transplant program outcomes compared to national benchmarks. Funnel plots demonstrating center‐specific 1‐year standardized graft mortality ratios (SMR) for NMP cases can be seen in Figure S1. While most centers fell within two standard deviations of their expected failure ratio, six centers (Centers A‐F) deviated significantly above this norm. Notably, these centers were at the wider mouth of the funnel, with a lower number of expected events (i.e., smaller volume and/or lower risk profile). To examine if these findings were due to the learning curve with NMP adoption, the study period was divided into three sub‐eras (Era 1: January 10, 2021–December 31, 2022, Era 2: January 1, 2023–December 31, 2023, and Era 3: January 1, 2024–December 31, 2024). Across these periods, individual center NMP caseload increased, as did the number of centers utilizing NMP (Figure S2). In each era there were several outliers demonstrating significantly higher SMRs exceeding the 95% limits of the plot. Only one center (Center A) was a persistent outlier across two consecutive eras.

Recipients at the six outlying centers with higher than expected NMP graft failure rates over the study period were compared to recipients at all other centers (Table S1); 1‐year NMP graft failure rates were expectedly higher, at 17.3% at outlier centers compared to 7.5% at all others, as were 1‐year graft failure rates for all center recipients (11.5% vs. 7.8%, *p* < 0.001). Median yearly center volume was higher at high outlier centers (121.7 [88.9–163.7] transplants per year vs. 54.7 [18.2–105.8], *p* < 0.01), though NMP volumes did not significantly differ (16.5 [4.6–22.8] transplants per year vs. 7.1 [1.8–20.6], *p* = 0.29). These high outlier centers transplanted older patients (60 years [52–66.5] vs. 58 [49–65], *p* < 0.01) with lower MELD scores [18 [11, 12, 13, 14, 15, 16, 17, 18, 19, 20, 21, 22, 23] vs. 21 [15, 16, 17, 18, 19, 20, 21, 22, 23, 24, 25, 26, 27, 28], *p* < 0.001], who more likely had diagnoses of malignancy (23.2% vs. 18.3%) compared to recipients at all other centers. Recipients from centers with higher than expected graft failure rates also had higher functional status (Karnofsky score 80%+: 41.1% vs. 30.5%, *p* < 0.001), lower rates of pretransplant ICU admission (6.7% vs. 11.8%, *p* < 0.001), and more often had a history of PVT (21.7% vs. 15.0%, *p* < 0.01) or TIPS (16.7% vs. 12.1%, *p* = 0.02). Their grafts came from older donors (52 [37–61] vs. 48 [35–58.5], *p* < 0.001) with higher rates of hypertension (51.5% vs. 44.6%, *p* = 0.02) and underwent longer preservation times (17.0 [14.1–20.3] vs. 15.0 [11.3–19.1], *p* < 0.001).

## 4 Discussion

In the first 3 years of NMP use in the United States, grafts preserved using NMP had a 1‐year failure rate of 8.1%, identical to the SCS rate. Within this cohort, DCD NMP grafts had a significantly lower risk of failure than DBD NMP grafts through the first year posttransplant. Recipients with one‐year graft failure were clinically sicker at the time of transplant, characterized by lower Karnofsky functional scores and higher rates of ICU admission or hospitalization. In addition, they more often had histories of prior abdominal surgery, TIPS, or a prior liver transplant contributing to surgical complexity. While no donor‐specific characteristic appeared to negate the utility of NMP, careful recipient selection remains critical for maximizing benefit. Center‐level analysis revealed high failure rates at a small number of higher‐volume programs, partially explained by their lower‐risk recipient profiles, reinforcing the pivotal role of provider judgment and institutional risk assessment in determining patient outcomes.

Historical predictors of donor risk such as donor age and DCD status [18, 19] were not independently predictive of graft failure across any of the cohorts, reflecting the benefits of NMP in both graft rehabilitation and viability assessment. Furthermore, DCD grafts preserved with NMP demonstrated superior early graft survival compared to DBD grafts within this cohort. Selection bias may play a role in this observation as DBD grafts preserved with NMP may more often represent extended criteria donors in whom NMP was employed for donor‐related risk factors and/or transplantation into higher risk recipients. Overall, however, the lack of additional predictive donor characteristics provides further evidence that NMP mitigates many of the traditional donor risk factors.

Indeed, complex recipient factors were stronger independent predictors of NMP graft failure than any donor characteristics. A MELD score of ≥ 35 or a history of prior abdominal surgery increased the odds of failure by 110% and 33%, respectively. Prior abdominal surgery has consistently been identified as a negative prognostic factor for graft survival. OPTN data from 1987 to 2015 confirmed an independent association of prior upper abdominal surgery with poor outcomes in LT recipients with hepatocellular carcinoma [20], aligning with previous work defining abdominal reoperation as a marker of technically difficult transplantation [21, 22], characterized by greater blood loss, longer operative times, and increased transfusion requirements [22]. In the same study, ICU status pretransplant was likewise noted as a predictor of difficult LT [22], and an independent risk factor for DBD graft failure in our analysis. Interestingly, Ebaid et al. [23] found that in high acuity recipients, NMP improved intraoperative and postoperative outcomes compared with matched SCS controls, with a 6‐month mortality of only 6%. These findings suggest that transplant surgeons may be accepting higher recipient surgical risk given the improved control over donor quality and operative timing afforded by NMP. Nevertheless, despite the advantages of graft optimization, recipient‐related factors, particularly those reflecting surgical complexity and acuity, remain key determinants of transplant outcomes.

Given the heterogeneous use of NMP and variable liver acceptance criteria across transplant programs, significant center‐level variation in graft failure rates was anticipated; however, most centers tracked within expected ranges. We did not find evidence of widespread persistent center‐level underperformance, and apparent outliers were few and largely nonpersistent over time. The counterintuitive finding of poor outcomes in seemingly favorable cases of lower‐acuity recipients may illuminate critical gaps in clinical judgment surrounding NMP utilization. On examination of these six outlying centers, recipients appeared older with lower laboratory MELD scores, higher rates of malignancy, and better functional status. Combined with findings of older donors and larger center volumes, this pattern suggests aggressive attempts to expand transplant criteria using NMP technology, potentially in cases where traditional risk metrics inadequately captured true recipient complexity. These data underscore a fundamental challenge: standard acuity measures like MELD may not fully represent transplant risk in the NMP era, making clinical judgment even more paramount. The apparent disconnect between conventional low‐risk profiles and poor outcomes at these centers highlights how provider decision‐making and institutional risk assessment become critical determinants of success when pushing traditional boundaries. In the time series analysis, the absence of persistent outliers further suggests most programs successfully adapt to NMP integration without sustained performance deterioration, with transient deviations likely reflecting case mix fluctuations rather than structural care deficiencies. These findings support the overall reliability of NMP across diverse centers while emphasizing that institutional experience and nuanced recipient‐graft matching beyond conventional risk metrics are essential for optimizing outcomes in this evolving landscape.

The current cost has stifled widespread adoption of NMP for both DBD and DCD liver transplant as the standard of care, and careful consideration of resource utilization is therefore essential, particularly within healthcare systems constrained by limited budgets. Despite higher upfront organ acquisition and index hospitalization costs [24], however, multiple studies have found NMP to provide substantial clinical benefit, including reduced hospital resource use [11], shorter index hospital stays [11, 24], fewer posttransplant complications [25], and lower readmission rates [11]. Center NMP use has also been associated with lower health care costs for waitlisted candidates, potentially due to shorter waitlist times [25]. This demonstration of cost neutrality is critical for continued reimbursement [26]. However, the cost‐effectiveness of NMP must be evaluated within each healthcare system and national infrastructure, as economic pressures and stakeholders differ across settings [27]. The value of NMP persists beyond graft optimization to improving transplant logistics, including better control of transplant timing, increasing daytime case starts [24, 28], allowing acceptance of livers for multiple recipients, as well as functional assessment of a graft prior to implantation. Considerations of hospital resource utilization will remain central to defining the evolving role of NMP in clinical practice.

Within the limitations of our study lies the retrospective nature of the SRTR database, with particular concern for the accuracy of perfusion strategy classification. Our population may exclude a cohort of NMP transplants, namely those in which a back‐to‐base NMP strategy is employed, as such grafts may be misclassified given their initial preservation with SCS after procurement. Furthermore, normothermic regional perfusion cases are not explicitly denoted within the dataset, precluding adjustment for this important confounder. Beyond uncertainty in how NMP cases are classified lies an equally important gap: the absence of a documented rationale for why a given graft was placed on NMP in the first place. Separating cases of logistic convenience versus organ optimization could help narrow down true differences between NMP failures and successes. Similarly, no data are collected on graft perfusion parameters and biochemical viability testing performed while on NMP, nor granular decision‐making surrounding organ discard following viability assessment, preventing comprehensive cost‐effectiveness evaluation of NMP utilization. Published viability criteria focus on isolated perfusion parameters (perfusate and bile glucose, pH, transaminases, lactate clearance) [29] and their association with outcomes such as early allograft dysfunction [30], primary non‐function [31], and non‐anastomotic biliary strictures [2]. Incorporating donor and recipient factors, as described in this work, would permit the development of a dynamic framework for NMP utilization and graft implantation, one that current registry‐level data alone cannot fully establish.

In the context of donor optimization with NMP, adverse 1‐year outcomes cluster in recipients with frailty, pretransplant acuity, and technical complexity. Transplant provider clinical judgment remains a critical component of recipient‐donor matching and organ offer acceptance, which could help further optimize utilization of a scarce resource. Center‐level variation in NMP graft failure rates also warrants attention, as differences in practice patterns may influence outcomes and highlight opportunities for standardization and quality improvement, particularly in low‐volume programs or those with a low‐risk case mix.

## Author Contributions

L.E.M.: study conception and design, data analysis and interpretation, and manuscript drafting and critical revisions. A.K.M.: study design, data analysis and interpretation, manuscript drafting, and critical revisions. M.H., A.A., T.M.C., and S.M.: study design, data interpretation, manuscript drafting, and critical manuscript revisions. N.R., A.M., and P.A.V.: study design, data interpretation, and critical manuscript revisions. M.S.P.: study conception and design, data interpretation, manuscript drafting and critical revisions.

## Funding

Abdullah K. Malik is supported by a Clinical Research Training Fellowship from the Medical Research Council (MR/Z50502X/1). The funder had no role in design or conduct of the study; collection, management, analysis, and interpretation of the data; or manuscript preparation. Madhukar S. Patel is supported as the Dedman Scholar of Clinical Care at UT Southwestern Medical Center. The funder had no role in the design and conduct of the study; collection, management, analysis, and interpretation of the data; or preparation of the manuscript.

## Conflicts of Interest

The authors declare no conflicts of interest.

## Supporting information


**Table S1:** Selected characteristics of patients and grafts undergoing normothermic machine perfusion (NMP) transplant at centers with higher‐than‐expected rates of 1‐year graft failure (center *N* = 6), compared to those at other centers.
**Figure S1:** Funnel plots demonstrating center standardized graft mortality rates (SMR) for all normothermic machine perfusion (NMP) cases performed from January 10, 2021, to December 31, 2024. Reference line set at 1, indicating observed cases are equal to expected cases.
**Figure S2:** Funnel plots demonstrating center standardized graft mortality rates (SMR) for all normothermic machine perfusion (NMP) cases over time, from (A) January 1, 2021–December 31, 2022, (B) January 1, 2023–December 31, 2023, and (C) January 1, 2024–December 31, 2024. Reference line set at 1, indicating observed cases are equal to expected cases.

## Data Availability

The data reported here have been supplied by the Scientific Registry of Transplant Recipients as a contractor for the Organ Procurement and Transplantation Network and are available at https://www.srtr.org/reports/program‐specific‐reports/. The interpretation and reporting of these data are the responsibility of the authors and in no way should be seen as an official policy of or interpretation by the OPTN or the U.S. Government.
