# Supplementary material for: When Machine Perfusion Falls Short: Identifying Risks of Liver Graft Failure After Normothermic Machine Perfusion
Source: Artif Organs. 2026 Jul 18;50(10):1407–17. doi: 10.1111/aor.70195 (PMC13622930; doi:10.1111/aor.70195)
Supplement: Supplementary file 1 [file AOR-50-1407-s001.docx]

**Supplemental Table 1**. Selected characteristics of patients and grafts undergoing normothermic machine perfusion (NMP) transplant at centers with higher-than-expected rates of 1-year graft failure (center N=6), compared to those at other centers

|  | **All other**  **N=4,628** | **High outliers**  **N=300** | **Total**  **N=4,928** | **P value** |
| --- | --- | --- | --- | --- |
| Median yearly center volume^ | 54.7 [18.2-105.8] | 121.7 [88.9-163.7] | 60.9 [19.7-106.8] | **<0.01** |
| Median yearly center NMP volume^ | 7.1 [1.8-20.6] | 16.5 [4.6-22.8] | 7.1 [2.2-20.6] | 0.29 |
| 1-year overall graft failure | 2,161 (7.8%) | 281 (11.5%) | 2,442 (8.1%) | **<0.001** |
| 1-year NMP graft failure | 346 (7.5%) | 52 (17.3%) | 398 (8.1%) | **<0.01** |
| Recipient age (years) | 58 [49-65] | 60 [52-66.5] | 58 [49-65] | **<0.01** |
| Recipient sex (male) | 2,897 (62.6%) | 200 (66.7%) | 3,097 (62.8%) | 0.16 |
| Recipient lab MELD | 21 [15-28] | 18 [11-23] | 21 [15-28] | **<0.001** |
| Primary diagnosis |  |  |  | **<0.001** |
| MASLD | 949 (21.0%) | 43 (14.7%) | 992 (20.6%) |  |
| ALD | 1,533 (33.8%) | 82 (28.0%) | 1,615 (33.5%) |  |
| Viral | 208 (4.6%) | 17 (5.8%) | 225 (4.7%) |  |
| Malignancy | 830 (18.3%) | 68 (23.2%) | 898 (18.6%) |  |
| Cholestatic | 315 (7.0%) | 17 (5.8%) | 332 (6.9%) |  |
| Metabolic | 85 (1.9%) | 1 (0.3%) | 86 (1.8%) |  |
| Autoimmune | 113 (2.5%) | 6 (2.0%) | 119 (2.5%) |  |
| Unknown/cryptogenic | 159 (3.5%) | 15 (5.1%) | 174 (3.6%) |  |
| Other | 337 (7.4%) | 44 (15.0%) | 381 (7.9%) |  |
| Time on waitlist (days) | 52 [12-212] | 61 [15.5-216.5] | 52 [12-213] | 0.18 |
| Karnofsky functional status |  |  |  | **<0.001** |
| 10-40% | 983 (21.6%) | 54 (18.1%) | 1,037 (21.4%) |  |
| 50-70% | 2,174 (47.8%) | 119 (39.8%) | 2,293 (47.3%) |  |
| 80%+ | 1,387 (30.5%) | 126 (42.1%) | 1,513 (31.2%) |  |
| Admission status at transplant |  |  |  | **<0.001** |
| ICU | 546 (11.8%) | 20 (6.7%) | 566 (11.5%) |  |
| Hospitalized | 834 (18.0%) | 90 (30.0%) | 924 (18.8%) |  |
| Not hospitalized | 3,248 (70.2%) | 190 (63.3%) | 3,438 (69.8%) |  |
| Prior liver transplant | 191 (4.1%) | 5 (1.7%) | 196 (4.0%) | **0.04** |
| Portal venous thrombus | 693 (15.0%) | 65 (21.7%) | 758 (15.4%) | **<0.01** |
| Prior abdominal surgery | 2,521 (55.4%) | 168 (56.0%) | 2,689 (55.5%) | 0.85 |
| TIPS | 536 (12.1%) | 50 (16.7%) | 586 (12.4%) | **0.02** |
| Donor age (years) | 48 [35-58.5] | 52 [37-61] | 48 [35-59] | **<0.001** |
| Donor BMI (kg/m^2^) | 28.3 [24.2-33.3] | 28.1 [24.2-33.4] | 28.3 [24.2-33.3] | 0.86 |
| DCD donor | 2,663 (57.5%) | 158 (52.7%) | 2,821 (57.2%) | 0.10 |
| Terminal AST | 45 [26-81.5] | 46 [27-89] | 45 [26-82] | 0.27 |
| Terminal ALT | 36 [21-77.5] | 37 [21-72] | 36 [21-77.5] | 0.64 |
| Terminal total bilirubin | 0.6 [0.4-0.9] | 0.6 [0.4-0.9] | 0.6 [0.4-0.9] | 0.71 |
| Preservation time (CIT, hours) | 15.0 [11.3-19.1] | 17.0 [14.1-20.3] | 15.2 [11.5-19.2] | **<0.001** |
| Macro fat (%) | 5 [0-10] | 5 [0-10] | 5 [0-10] | 0.64 |
| Donor diabetes | 799 (17.5%) | 63 (21.2%) | 862 (17.8%) | 0.12 |
| Donor HTN | 2,031 (44.6%) | 154 (51.5%) | 2,185 (45.1%) | **0.02** |

**MELD**: Model for End Stage Liver Disease; **MASLD**: Metabolic dysfunction-associated steatotic liver disease; **ALD**: alcohol-associated liver disease; **ICU**: intensive care unit; **TIPS**: transjugular intrahepatic portosystemic shunt; **BMI**: body mass index; **DCD**: donation after circulatory death; **CIT**: cold ischemia time; **HTN**: hypertension

^Total study volume normalized to a 12-month period

**Supplemental Figure 1**. Funnel plots demonstrating center standardized graft mortality rates (SMR) for all normothermic machine perfusion (NMP) cases performed from 10/1/2021-12/31/24. Reference line set at 1, indicating observed cases are equal to expected cases.

**Supplemental Figure 2ABC**. Funnel plots demonstrating center standardized graft mortality rates (SMR) for all normothermic machine perfusion (NMP) cases over time, from (A) 10/1/2021-12/31/2022, (B) 1/1/2023-12/31/2023, and (C) 1/1/2024-12/31/2024. Reference line set at 1, indicating observed cases are equal to expected cases.
